# Development and validation of an AMR-based predictive model for post-PCI contrast-induced nephropathy in patients with acute ST-segment elevation myocardial infarction

**DOI:** 10.3389/fcvm.2025.1552762

**Published:** 2025-06-26

**Authors:** Zhaokai Wang, Shuping Yang, Cheng Li, Chunxue Zhou, Chaofan Wang, Tangxing Jiang, Chengcheng Chen, Mengxin Shao, Tongda Xu

**Affiliations:** ^1^ Department of Cardiology, The Affiliated Hospital of Xuzhou Medical University, Xuzhou, Jiangsu, China; ^2^ Department of General Practice, The Affiliated Hospital of Xuzhou Medical University, Xuzhou, Jiangsu, China

**Keywords:** nomogram, percutaneous coronary intervention, contrast-induced nephropathy, acute ST-segment elevation myocardial infarction, angiography-derived microcirculatory resistance index, prediction model

## Abstract

**Background:**

This study aimed to develop and validate an angiography-derived microcirculatory resistance index (AMR)- based nomogram to predict the probability of contrast-induced nephropathy (CIN) following percutaneous coronary intervention (PCI) in patients with acute ST-segment elevation myocardial infarction (STEMI).

**Method:**

In this study, 595 STEMI patients from the Affiliated Hospital of Xuzhou Medical University from January 1, 2022 to December 31, 2023 were included as the training cohort, and 256 patients from the East Hospital of Xuzhou Medical University were included as the validation cohort. Independent risk factors for the development of nomogram were identified using univariate logistic regression, randomized forest regression, multifactorial logistic regression, and LASSO regression analyses. The study evaluated performance by creating calibration curves, analyzing the area under the curve (AUC-ROC) of subjects' work characteristics, examining calibration plots, and conducting decision curve analysis (DCA).

**Result:**

Multifactorial logistic regression analysis identified five independent predictors, including eGFR (OR:0.975; 95% CI: 0.970–0.983; *P* < 0.001), AMR (OR: 2.505; 95% CI: 1.756–3.656; *P* < 0.001), Serum blood uric acid to high-density lipoprotein cholesterol ratio (UHR) (OR: 1.006; 95% CI: 1.003–1.007; *P* < 0.001), The triglyceride and glucose index (TyG) (OR: 1.829; 95% CI: 1.346–2.502; *P* < 0.001), Contrast agent dosage (OR: 1.022; 95% CI: 1.016–1.028; *P* < 0.001), The nomogram accurately predicted the probability of CIN after PCI. Both the training cohort (AUC: 0.881) and validation cohort (AUC: 0.841) demonstrated good predictive ability of the model. Calibration plots confirmed the agreement between the predictions of the training and validation cohorts. DCA analysis also demonstrated the feasibility of the nomogram in clinical patient management.

**Conclusion:**

The nomogram showed good performance in predicting CIN, and it could help clinicians optimize the clinical treatments to improve the prognosis of STEMI patients.

## Introduction

Acute ST-segment elevation myocardial infarction (STEMI) is a life-threatening cardiovascular emergency that requires prompt reperfusion therapy to restore blood flow and minimize myocardial damage (1). Percutaneous coronary intervention (PCI) is the cornerstone of STEMI treatment, providing a way to restore blood flow quickly and effectively. However, iodinated contrast agents used during PCI, while critical for visualizing coronary anatomy, are associated with the development of contrast-induced nephropathy (CIN), an important and potentially serious complication characterized by acute deterioration of renal function after exposure to contrast (2). CIN not solely complicates the immediate postoperative course of treatment, but also significantly affects the long-term prognosis of patients, including increased morbidity and mortality (3).

CIN is a serious complication that can occur following the administration of iodinated contrast agents during PCI. The incidence of CIN in STEMI patients after PCI has been reported to be 10%–13% and is associated with prolonged hospitalization, increased risk of adverse cardiovascular events, and increased mortality (4, 5). Given the high prevalence of CIN and its far-reaching implications, predictive models are urgently needed to identify patients at high risk of developing CIN after PCI.

In previous studies, several patient- and procedure-related risk factors associated with CIN have been identified, including advancing age, diabetes mellitus, prior renal dysfunction, anemia, hypertension, and contrast dose (6). Studies have shown that with increasing age, renal function progressively decreases, placing the elderly population at a significantly increased risk of CIN when undergoing contrast imaging (7). In addition, diabetic patients have impaired microvascular function and the effects of hyperglycemia, making them more likely to develop CIN after undergoing contrast (8). It is worth noting that although many studies have revealed these risk factors, the scope of most studies is usually limited to specific disease types, medications, and physiologic conditions, and there is a lack of comprehensive studies of broader populations. Emerging evidence suggests that impaired renal microvascular function may play a crucial role in the pathogenesis of CIN, and probably is associated with a higher risk of CIN development in STEMI patients, but the exact pathophysiologic mechanisms are unclear (9, 10). Evidence suggests that impairment of coronary microvascular function may affect renal perfusion, thereby increasing the risk of CIN in patients with STEMI. For example, it has been found that pathological changes in the coronary microcirculation are closely related to the function of the renal microcirculation, which may contribute to the development of CIN by affecting renal hemodynamics leading to inadequate microcirculatory perfusion (11). To assess coronary microcirculation, commonly used methods include invasive techniques (e.g., index of microcirculatory resistance, IMR, and flow reserve fraction, FFR) as well as noninvasive methods (e.g., ultrasonography and cardiac magnetic resonance imaging, CMR). Recently, in the realm of coronary microcirculation, a novel concept has been introduced—the angiography-derived index of microcirculatory resistance (AMR). This approach is different from the traditional index of microcirculatory resistance (IMR) measurement technique by offering a non-invasive, cost-effective, quantitative assessment method that eliminates the need for expensive pressure wires, vasodilatory drugs, or any additional surgical interventions, and automatically produces results from a single coronary angiographic image. Research has demonstrated that the AMR has excellent concordance and accuracy with the IMR in predicting microvascular function, and that elevated AMR predicts the presence of microvascular obstruction in CMR (12, 13). Given that AMR reflects cardiac microcirculation, we hypothesize that damage to the cardiac microcirculation may predict renal microcirculatory insufficiency, thereby serving as a valuable tool in anticipating renal damage in CIN. Furthermore, the relationship between AMR and CIN has not been adequately explored in the existing literature, highlighting the novelty and significance of our study. By focusing on the role of AMR in predicting CIN among STEMI patients, we aim to provide new insights into the preventive strategies for renal impairment in this high-risk population.

The nomogram clinical prediction model can quantitatively calculate the incidence of events. Due to its intuitively comprehensible nature and high accuracy, the nomogram has extensive applications in medical staging, treatment strategies, and patient management. The objective of this study is to utilize clinical data from Chinese patients to develop a risk prediction chart for post-PCI CIN in patients with STEMI. This approach aims to identify high-risk patients, conduct risk stratification, ultimately reduce morbidity, and improve postoperative quality of life.

## Material and methods

### Study population and design

**Figure 1:**
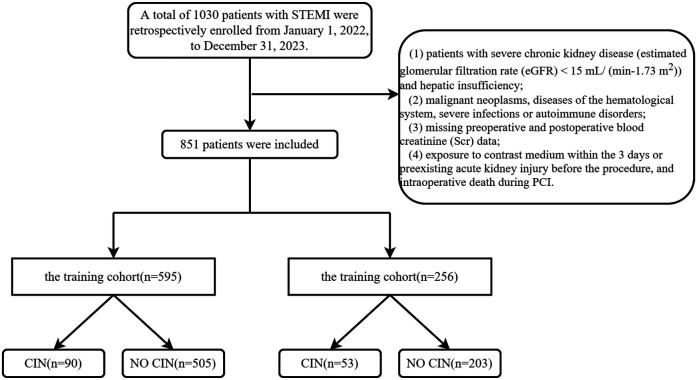
Flow chart. STEMI, acute st-segment elevation myocardial infarction; CIN, contrast-induced nephropathy.

This study (XYFY2023-KL043-01) was conducted following the Declaration of Helsinki and approved by the Medical Research Ethics Committee of the Affiliated Hospital of Xuzhou Medical University. Because our study was retrospective, the committee waived the requirement for written informed consent. Based on the inclusion and exclusion criteria, we included patients with NSTEMI who underwent PCI from January 1, 2022, to December 31, 2023, at the Affiliated Hospital of Xuzhou Medical University (training group, *n* = 595) and the East Hospital of Xuzhou Medical University (validation group, *n* = 256). Exclusion criteria for the study included patients with severe chronic kidney disease [estimated glomerular filtration rate (eGFR) < 15 ml/ (min−1.73 m^2^)] and hepatic insufficiency, malignant neoplasms, diseases of the hematological system, severe infections or autoimmune disorders, missing preoperative and postoperative blood creatinine (Scr) data, exposure to contrast medium within the 3 days or preexisting acute kidney injury before the procedure, and intraoperative death during PCI. (Figure 1).

### In-hospital treatment procedure

All patients with STEMI were sent to the digital subtraction angiography (DSA) room for PCI, where patients were anticoagulated with heparin subcutaneously and given 300 mg of aspirin, 300 mg of clopidogrel, or 180 mg of Ticagrelor orally as a loading dose before coronary angiography or PCI. The various devices, instruments, and adjunctive medications (nitroglycerin, sodium nitroprusside, tirofiban, atropine, etc.) used during the procedure are determined by the operator's assessment and the patient's intraoperative condition. Postoperatively, a routine drug regimen such as aspirin (100 mg qd) + clopidogrel (75 mg qd) or aspirin (100 mg qd) + Ticagrelor (90 mg bid), both in combination for at least 1 year, and statins should be administered. A nonionic isotonic osmotic contrast agent (ioversol, Yangtze River Pharmaceutical Group) was used during the procedure with an osmolality concentration of 800 mOsm/Kg. The interventionalist determined the dosage during the procedure based on the patient's condition. After PCI, all images are copied and analyzed using the quantitative blood flow scoring device from Pulse Medical to obtain the AMR value of the culprit's blood vessels (14).

### Assessment of AMR

AMR analysis was performed independently by certified technicians using commercial software (AngioPlus Core, Pulse Medical Imaging Technology Co., Ltd., Shanghai, China), who were blinded to the clinical data. Coronary artery image analysis was performed using the above system. The blood flow velocity was derived by dividing the vessel centerline length by the contrast fill time. Using high blood flow as a boundary condition, the pressure drop was calculated from the hydrodynamic equations. Distal coronary pressure (Pd) was calculated from the pressure drop, and μQFR was calculated by dividing Pd by mean aortic pressure (Pa). Angiography microvascular resistance (AMR) is computed as Pd divided by the hyperaemic flow velocity Velocity_hyp_ (15).

$$\mathrm{AMR}=\frac{P_{d}}{\mathrm{Velocit}y_{hyp}}=\frac{P_{a}\;*\;\mu \mathrm{QFR}}{\mathrm{Velocit}y_{hyp}}$$

## Definitions

CIN is defined as: Excluding other causes, an increase in serum creatinine levels by at least 0.5 mg/dl (44 μmol/L) or more than 25% above baseline within 2–3 days after the use of a contrast agent. Related definitions and diagnostic criteria included the following: Chronic kidney disease (CKD) is defined as an estimated glomerular filtration rate (eGFR) of less than 60 ml/min/1.73 m^2^ (16, 17). NLR is defined as the ratio of neutrophil count to lymphocyte count. The triglyceride-glucose index was calculated as TyG = ln [fasting triglyceride (mg/dl) × fasting glucose (mg/dl)]/2 (18). UHR was defined as the ratio of serum blood uric acid to high-density lipoprotein cholesterol (19).

### Collection of data

Clinical data collection information, including demographics, laboratory parameters, physical examination data, and related indicators, were collected through the electronic medical record. Demographic information included age, gender, hypertension, diabetes, history of smoking (Smoke), and CKD. Physical examination included heart rate (HR), systolic blood pressure (SBP), and diastolic blood pressure (DBP), Killip grade. Laboratory tests included leucocyte (WBC), neutrophil count, lymphocyte count, monocyte count, hemoglobin (Hb), erythrocyte distribution width (RDW), platelet (PLT) count, platelet distribution width (PDW), mean platelet volume (MPV), high sensitive c-reactive protein (hs-CRP), fibrinogen (FIB), lactate dehydrogenase (LDH), lactate dehydrogenase (LDH), creatine kinase (CK), creatine kinase-mb (CK-MB), high-sensitivity troponin t (hs-TnT), n-terminal pro-brain natriuretic peptide (NTpro-BNP), Prealbumin, blbumin, blood urea nitrogen (BUN), cystatin c (Cys C), eGFR, total bilirubin (TBIL), direct bilirubin (DBIL), total cholesterol (TC), triglycerides (TG), high-density lipoprotein (HDL), low-density lipoprotein (LDL), lipoprotein a (LPa), and small dense low-density lipoprotein-cholesterol (sdLDL-C), TyG, neutrophil-to-lymphocyte Ratio (NLR), serum blood uric acid to high-density lipoprotein cholesterol ratio (UHR), triglycerides to high-density lipoprotein cholesterol ratio (TG/HDL-C), and all baseline laboratory parameters were the latest test results before PCI. The treatment strategy included the surgical duration, culprit vessel, AMR of culprit vessel, contrast agent dosage, and the number of stents. All endpoint events were adjudicated centrally by two independent cardiologists, and disagreements were resolved by consulting a third expert.

## Statistical analysis

Continuous variables were expressed as mean ± standard deviation (*x* ± s), tested for normality using the chi-square test, and a *t*-test was used for normally distributed variables. Otherwise, the Mann–Whitney *U*-test was employed. Categorical variables were expressed as frequencies plus percentages and compared using the chi-square test or Fisher's exact calculation method. This study employed univariate logistic regression analysis and random forest regression to identify significant predictors of CIN after PCI. In this study, random forest regression generates the mean Gini coefficient (MDG) of the respective variables. This reflects the contribution of the variables to the risk of post-PCI CIN and explains the relationship between the independent and dependent variables. This method is more resistant to interference than the traditional method and complements the results of the complementary one-way logistic regression analysis, making the results more accurate. The analysis will include variables that are statistically significant in univariate analysis (*P* < 0.05) and the top 50% of independent variables in random forest regression. The identified significant factors will be used to perform multivariate logistic regression models. To avoid overfitting, Lasso regression will be used to eliminate highly correlated factors. Finally, a nomogram predicting the probability of CIN after PCI is constructed based on a multivariate model consisting of the optimal predictors. Nomogram evaluation included discriminatory ability, calibration, and clinical effectiveness. The discrimination capacity of the nomogram was quantified by measuring the consistency index (C-index), which was equal to the area under the subject working characteristic curve (AUC-ROC) in the logistic regression analysis. The predictive accuracy of the nomograms was evaluated using calibration plots, and the Hosmer-Lemeshow test was conducted to assess the consistency between the predicted values and the actual probabilities. Additionally, decision curve analysis (DCA) was employed to assess the clinical effectiveness of the nomograms. The data analysis was conducted using R Studio (Version 4.2.3, https://www.Rproject.org). All statistical tests were considered significant for *p* values < 0.05.

## Results

### Characteristics of the study population

Clinical data were collected from a total of 1030 study subjects. Our study included 851 STEMI patients after PCI from January 1, 2022, to December 31, 2023, from the Affiliated Hospital of Xuzhou Medical University and the East Affiliated Hospital of Xuzhou Medical University. The mean age in this study was 67 years, and most of them were male. The baseline characteristics of the two cohorts were not significantly different and were comparable (Table 1).

**Table 1:** Patient characteristics.

Variables	Validation cohort (*N* = 256)	Training cohort (*N* = 595)	*P* value
Age, years	67 (55.75–73.00)	67 (54.50–74.00)	0.389
Gender (%)			
Female, *n* (%)	63 (24.6)	137 (23.0)	0.617
Male, *n* (%)	193 (75.4)	458 (77.0)	
SBP, mmHg	129 (113.00–142.00)	128 (112.00–140.00)	0.358
DBP, mmHg	79 (70.00–87.25)	78 (70.00–87.00)	0.370
HR, times/min	78 (69–86)	78 (69–89)	0.452
Smoking, *n* (%)	113 (44.1)	269 (45.2)	0.774
Hypertension, *n* (%)	119 (46.5)	274 (46.1)	0.907
Diabete, *n* (%)	90 (35.2)	209 (35.1)	0.993
CKD, *n* (%)	31 (12.1)	59 (9.9)	0.340
Laboratory test			
WBC, ×10^9^/L	9.90 (8.70–12.30)	9.70 (8.10–11.90)	0.196
N, ×10^9^/L	7.63 (5.84–9.35)	7.08 (5.45–9.30)	0.072
L, ×10^9^/L	1.40 (1.10–2.30)	1.50 (1.10–2.30)	0.488
M, ×10^9^/L	0.54 (0.38–0.76)	0.52 (0.40–0.74)	0.912
Hb, g/L	141.00 (126.00–153.00)	142.00 (127.00–154.00)	0.518
RBC, ×10^9^/L	4.62 (4.11–4.94)	4.61 (4.15–4.97)	0.754
RDW, fL	42.30 (37.92–45.60)	42.20 (38.20–45.70)	0.581
PLT, ×10^9^/L	206.50 (170.00–253.50)	206.00 (171.00–244.00)	0.927
PDW, fL	13.45 (10.88–16.10)	13.50 (11.00–16.20)	0.546
MPV	10.00 (9.40–10.90)	10.00 (9.40–10.80)	0.801
hs-CRP, mg/L			
≤11	190 (74.2)	464 (78.0)	0.232
≥11	66 (25.8)	131 (22.0)	
FIB, g/L	2.85 (2.33–3.67)	2.80 (2.32–3.55)	0.855
LDH, U/L	530.50 (358.00–763.50)	515.00 (347.00–740.00)	0.433
CK, U/L	155.00 (62.75–605.00)	159.00 (70.00–492.50)	0.812
CKMB, U/L	11.65 (3.79–37.26)	9.92 (2.90–45.17)	0.563
hsTnT, ng/L	128.50 (31.69–707.45)	175.00 (34.33–690.60)	0.558
NT-proBNP, pg/ml	774.00 (163.41–1,959.50)	774.00 (166.10–2,109.00)	0.905
Prealbumin, g/L	0.23 (0.19–0.26)	0.23 (0.19–0.26)	0.996
Albumin, g/L	38.40 (35.77–41.20)	38.60 (36.10–41.40)	0.195
BUN, mmol/L	6.06 (5.00–7.74)	5.86 (4.84–7.44)	0.201
Cycs, mg/L	0.88 (0.77–1.00)	0.87 (0.74–0.99)	0.248
eGFR, ml/min/1,73 m^2^	117.26 (101.37–142.26)	125.64 (101.78–150.55)	0.058
Glucose, mmol/L	5.92 (5.09–7.98)	5.88 (5.10–8.01)	0.975
TBIL, umol/L	13.25 (9.70–18.60)	13.90 (9.90–19.00)	0.294
DBIL, umol/L	4.90 (3.90–6.80)	5.10 (4.00–6.80)	0.385
TC, mmol/L	4.28 (3.60–4.67)	4.22 (3.62–4.70)	0.860
TG, mmol/L	1.24 (0.93–1.75)	1.25 (0.90–1.86)	0.870
HDL, mmol/L	0.94 (0.83–1.12)	0.94 (0.82–1.12)	0.863
LDL, mmol/L	2.74 (2.23–3.08)	2.73 (2.16–3.11)	0.932
LPa, mg/L	203.50 (127.00–298.00)	214.00 (138.00–313.00)	0.324
Sd-LDL, mmol/L	0.74 (0.54–1.10)	0.76 (0.54–1.11)	0.980
TyG	9.00 (8.50–9.80)	9.00 (8.50–9.70)	0.685
NLR	5.20 (2.90–8.45)	4.90 (2.60–7.90)	0.230
TG/HDL-C	1.44 (0.86–1.95)	1.38 (0.86–2.06)	0.917
UHR	334.00 (229.25–428.00)	312.00 (214.50–419.00)	0.062
Angiographic features			
Killip grade			
Grade I, *n* (%)	251 (98.0)	586 (98.5)	0.831
Grade II, *n* (%)	3 (1.2)	5 (0.8)	
Grade III, *n* (%)	0 (0.0)	1 (0.2)	
Grade IV, *n* (%)	2 (0.8)	3 (0.5)	
Culprit Vessel			
LAD, *n* (%)	121 (47.3)	277 (46.6)	0.943
LCX, *n* (%)	52 (20.3)	118 (19.8)	
RCA, *n* (%)	83 (32.4)	200 (33.6)	
Contrast dosage, ml	165.00 (120.00–200.00)	160.00 (120.00–200.00)	0.325
Surgical duration,min	55.00 (45.00–70.00)	59.00 (48.00–70.00)	0.403
Number of stents, *n* (%)	1.00 (1.00–2.00)	1.00 (1.00–2.00)	0.841
AMR of culprit vessel	2.32(1.72–2.72)	2.28(1.67–2.70)	0.455

SBP, systolic blood pressure; DBP, diastolic blood pressure; HR, heart rates, CKD, chronic kidney disease; Hb, hemoglobin; WBC, white blood cell; N, neutrophils; L, lymphocyte; M, monocyte; Hb, hemoglobin; RBC, red blood cell; RDW, erythrocyte distribution width; PLT, platelet; PDW, platelet distribution width; MPV, mean platelet volume; hs-CRP, high sensitive c-reactive protein; FIB, fibrinogen; LDH, lactate dehydrogenase; CK, creatine kinase; CKMB, creatine kinase isoenzyme MB; hs-TnT, high-sensitivity troponin t; NTpro-BNP, n-terminal pro-brain natriuretic peptide; BUN, blood urea nitrogen; CysC, cystatin C; eGFR, estimated glomerular filtration rate; TBIL, total bilirubin; DBIL, direct bilirubin; TC, total cholesterol; TG, triglycerides; HDL, high-density lipoprotein; LDL, low-density lipoprotein; LP a, lipoprotein a; sdLDL-C, small dense low-density lipoprotein-cholesterol; TyG, the triglyceride and glucose index; NLR, neutrophil to lymphocyte ratio; TG/HDL-C, triglycerides to high-density lipoprotein cholesterol ratio; UHR, serum uric acid to high-density lipoprotein cholesterol ratio; AMR, angiography-derived microcirculatory resistance index.

### Predictors of CIN after PCI in patients with STEMI

**Figure 2:**
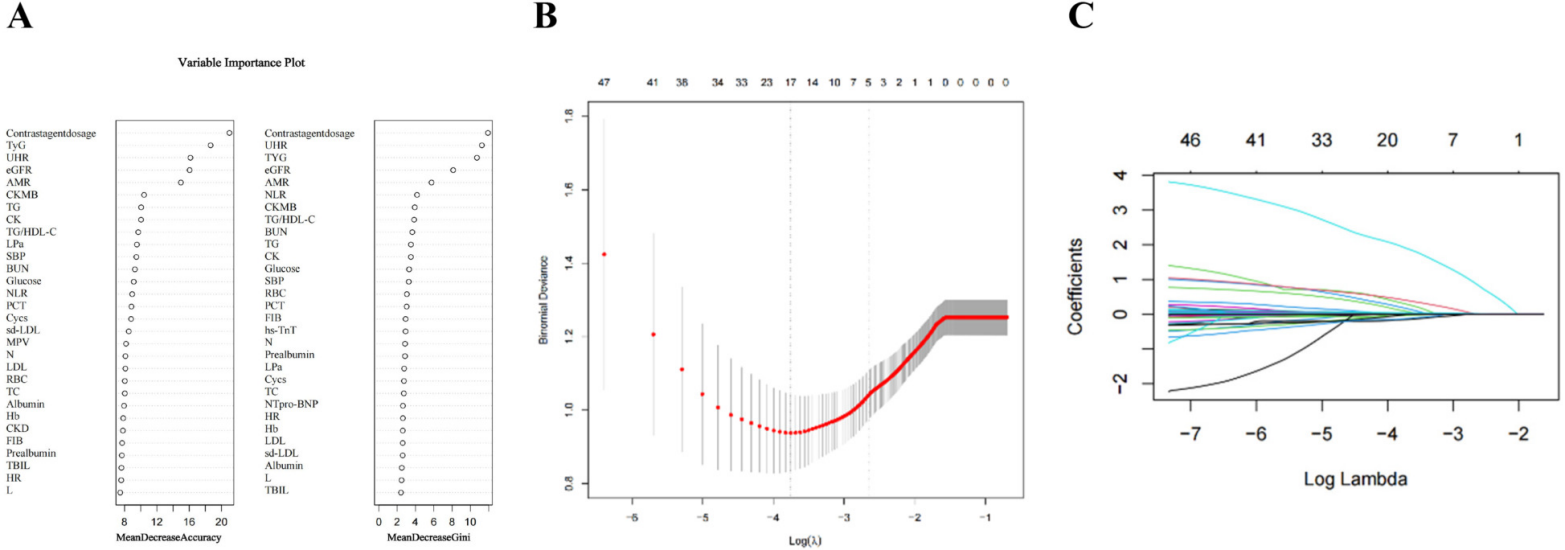
The variables filtering process of the lasso regression. To avoid overfitting, the Lasso regression suggests including 5 variables when CIN is the endpoint. In the variable selection process, potential CIN indicators were first selected using univariate logistic regression and variables in the top 50% of the Random Forest Variable Importance Score **(A)** Then, multivariate logistic regression models were constructed based on these potential indicators. In this study, lasso regression was used only to ensure that the multivariate logistic regression was not overfitted, not for variable selection and modeling. **(B)** Lasso coefficient curves for characteristics. **(C)** Optimal parameter (lambda) selection in the Lasso model with five-fold cross-validation by the minimum criterion.

Univariate regression analysis and randomized forest regression are shown in Tables 2 and Figure 2A, respectively. Twelve variables were statistically significant in the Univariate regression analysis, with TYG, AMR, eGFR, UHR, and contrast agent dosage yielding significant results not only in the Univariate regression analysis but also in the random forest. The above variables were included in a multifactorial logistic regression analysis to identify independent prognostic risk factors after PCI in patients with STEMI. Multifactorial regression showed that eGFR (OR:0.975; 95% CI: 0.970–0.983; *P* < 0.001), AMR (OR: 2.505; 95% CI: 1.756–3.656; *P* < 0.001), UHR (OR: 1.006; 95% CI: 1.003–1.007; *P* < 0.001), TyG (OR: 1.829; 95% CI: 1.346–2.502; *P* < 0.001), Contrast agent dosage (OR: 1.022; 95% CI: 1.016–1.028; *P* < 0.001) were independent risk factors (Table 3). The identification of independent predictive features within the training cohort was accomplished through the application of non-zero coefficients in Lasso regression. The optimal parameter selection (lambda) for the Lasso model was determined using five-fold cross-validation based on a minimum criterion. This approach helped mitigate the effects of multicollinearity, resulting in a model that demonstrated robust predictability and high stability (Figures 2B,C).

**Table 2:** Univariable logistic regression analysis for CIN in the training group.

Variables	OR	95%CI	*P* value
Age, years	1.013	(0.996–1.031)	0.125
Gender (%)			
Female, *n* (%)	Ref	Ref	–
Male, *n* (%)	0.793	(0.475–1.323)	0.125
SBP, mmHg	0.994	(0.983–1.006)	0.356
DBP, mmHg	0.993	(0.976–1.011)	0.442
HR, times/min	1.011	(0.996–1.027)	0.152
Smoking, *n* (%)	0.867	(0.551–1.364)	0.537
Hypertension, *n* (%)	1.270	(0.811–1.990)	0.296
Diabete, *n* (%)	0.808	(0.499,1.309)	0.387
CKD, *n* (%)	4.092	(2.278–7.351)	<0.0001
Laboratory test			
WBC, ×10^9^/L	1.043	(0.974–1.117)	0.225
N, ×10^9^/L	1.125	(1.051–1.204)	0.001
L, ×10^9^/L	0.600	(0.451–0.797)	<0.0001
M, ×10^9^/L	0.982	(0.484–1.994)	0.961
Hb, g/L	1.001	(0.989–1.013)	0.859
RBC, ×10^9^/L	1.082	(0.738–1.587)	0.658
RDW, fL	0.993	(0.953–1.035)	0.735
PLT, ×10^9^/L	1.000	(0.996–1.003)	0.926
PDW, fL	1.048	(0.965–1.138)	0.268
MPV	0.806	(0.648–1.004)	0.054
hs-CRP, mg/L			
≤11	Ref	Ref	–
≥11	1.353	(0.809–2.261)	0.249
FIB, g/L	1.093	(0.922–1.296)	0.307
LDH, U/L	1.000	(1.000–1.000)	0.749
CK, U/L	1.000	(1.000–1.000)	0.051
CKMB, U/L	1.004	(1.001–1.007)	0.003
hsTnT, ng/L	1.000	(1.000–1.000)	0.020
NT-proBNP, pg/ml	1.000	(1.000–1.000)	0.083
Prealbumin, g/L	0.045	(0.001–2.364)	0.125
Albumin, g/L	0.991	(0.939–1.046)	0.744
BUN, mmol/L	0.887	(0.793–0.992)	0.036
Cycs, mg/L	1.647	(0.702–3.861)	0.251
eGFR, ml/min/1.73 m^2^	0.978	(0.971–0.985)	<0.0001
Glucose, mmol/L	0.939	(0.863–1.023)	0.149
TBIL, μmol/L	1.016	(0.990–1.042)	0.225
DBIL, μmol/L	1.020	(0.965–1.078)	0.489
TC, mmol/L	1.030	(0.805–1.318)	0.814
TG, mmol/L	0.812	(0.611–1.079)	0.151
HDL, mmol/L	1.458	(0.547–3.889)	0.451
LDL, mmol/L	1.040	(0.787–1.375)	0.781
LPa, mg/L	1.000	(1.000–1.000)	0.123
Sd-LDL, mmol/L	0.668	(0.382–1.168)	0.157
TyG	1.825	(1.438–2.315)	<0.0001
NLR	1.109	(1.060–1.159)	<0.0001
TG/HDL-C	0.856	(0.692–1.058)	0.149
UHR	1.004	(1.003–1.006)	<0.0001
Angiographic features			
Killip grade			
Grade I, *n* (%)	Ref	Ref	–
Grade II, *n* (%)	1.453	(0.161–13.161)	0.739
Grade III, *n* (%)	3.150	(1.025–12.170)	0.977
Grade IV, *n* (%)	4.535	(0.406–50.687)	0.220
Culprit Vessel			
LAD, *n* (%)	Ref	Ref	–
LCX, *n* (%)	0.591	(0.307–1.137)	0.115
RCA, *n* (%)	0.809	(0.490–1.336)	0.408
Contrast agent dosage, ml	1.016	(1.012–1.021)	<0.0001
Surgical duration, min	1.004	(0.992–1.168)	0.498
Number of stents, *n* (%)	0.860	(0.539–1.371)	0.526
AMR of culprit vessel	1.755	(1.329–2.318)	<0.0001

SBP, systolic blood pressure; DBP, diastolic blood pressure; HR, heart rates, CKD, chronic kidney disease; Hb, hemoglobin; WBC, white blood cell; N, neutrophils; L, lymphocyte; M, monocyte; Hb, hemoglobin; RBC, red blood cell; RDW, erythrocyte distribution width; PLT, platelet; PDW, platelet distribution width; MPV, mean platelet volume; hs-CRP, high sensitive c-reactive protein; FIB, fibrinogen; LDH, lactate dehydrogenase; CK, creatine kinase; CKMB, creatine kinase isoenzyme MB; hs-TnT, high-sensitivity troponin t; NTpro-BNP, n-terminal pro-brain natriuretic peptide; BUN, blood urea nitrogen; CysC, cystatin C; eGFR, estimated glomerular filtration rate; TBIL, total bilirubin; DBIL, direct bilirubin; TC, total cholesterol; TG, triglycerides; HDL, high-density lipoprotein; LDL, low-density lipoprotein; LP a, lipoprotein a; sdLDL-C, small dense low-density lipoprotein-cholesterol; TyG, the triglyceride and glucose index; NLR, neutrophil to lymphocyte ratio; TG/HDL-C, triglycerides to high-density lipoprotein cholesterol ratio; UHR, serum uric acid to high-density lipoprotein cholesterol ratio; AMR, angiography-derived microcirculatory resistance index.

**Table 3:** Multivariate logistic regression analysis for CIN in the training group.

Variables	OR	95%CI	*P* value
eGFR	0.975	(0.970–0.983)	<0.001
AMR	2.505	(1.756–3.656)	<0.001
UHR	1.006	(1.003–1.007)	<0.001
TyG	1.829	(1.346–2.502)	<0.001
Contrast agent dasage	1.022	(1.016–1.028）	<0.001

eGFR, estimated glomerular filtration rate; AMR, angiography-derived microcirculatory resistance index; UHR, serum uric acid to high-density lipoprotein cholesterol ratio.

### Construction of the nomogram

**Figure 3:**
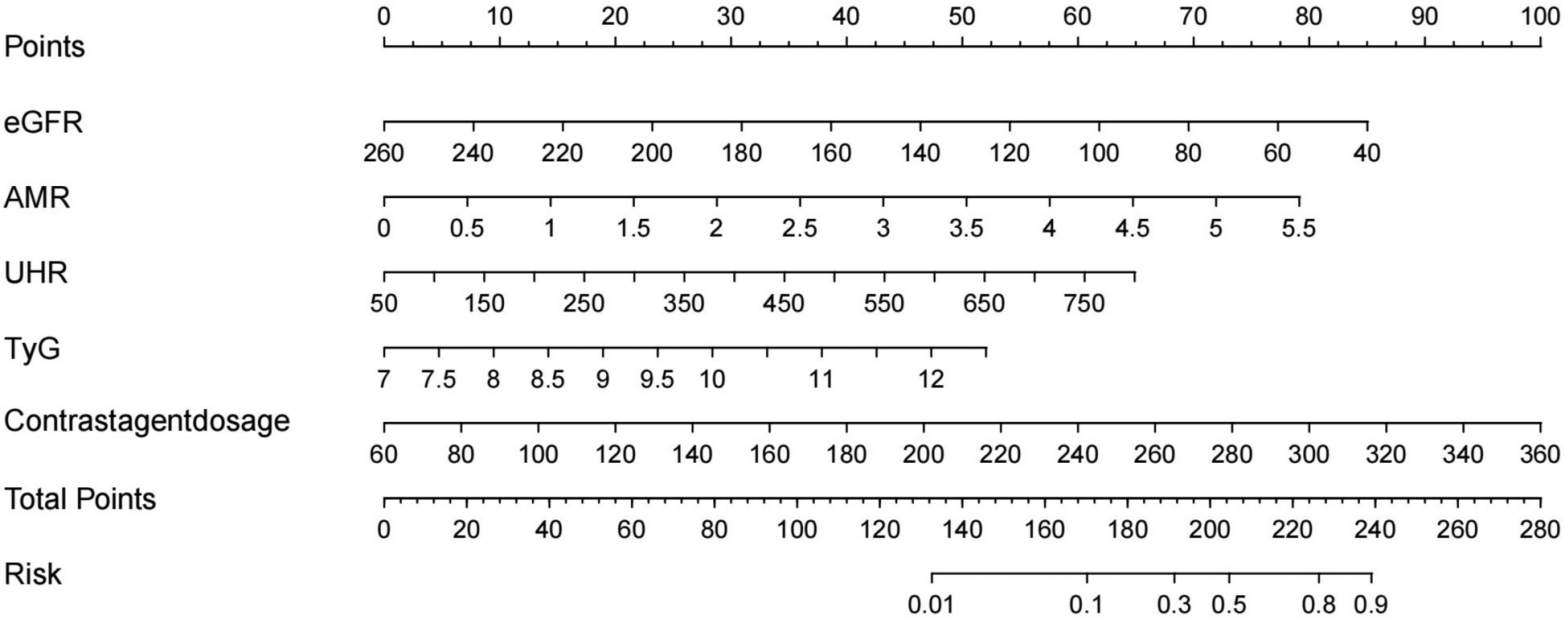
Nomogram for predicting the probability of CIN in patients with STEMI after PCI. eGFR, estimated glomerular filtration rate; AMR, angiography-derived microcirculatory resistance index; UHR, serum uric acid to high-density lipoprotein cholesterol ratio.

A nomogram model of five factors (eGFR, AMR, UHR, TYG, and Contrast agent dosage) associated with the occurrence of CIN after PCI in STEMI patients was developed based on univariate and multivariate logistic regression analysis. To determine the force of each covariate in the model, vertical lines were drawn on the scoring line at the top of the nomogram to obtain the corresponding points. The total score, which accurately predicts the probability of CIN occurrence after PCI in STEMI patients, can be calculated by adding the points of the five factors (Figure 3).

### Validation of the nomogram

**Figure 4:**
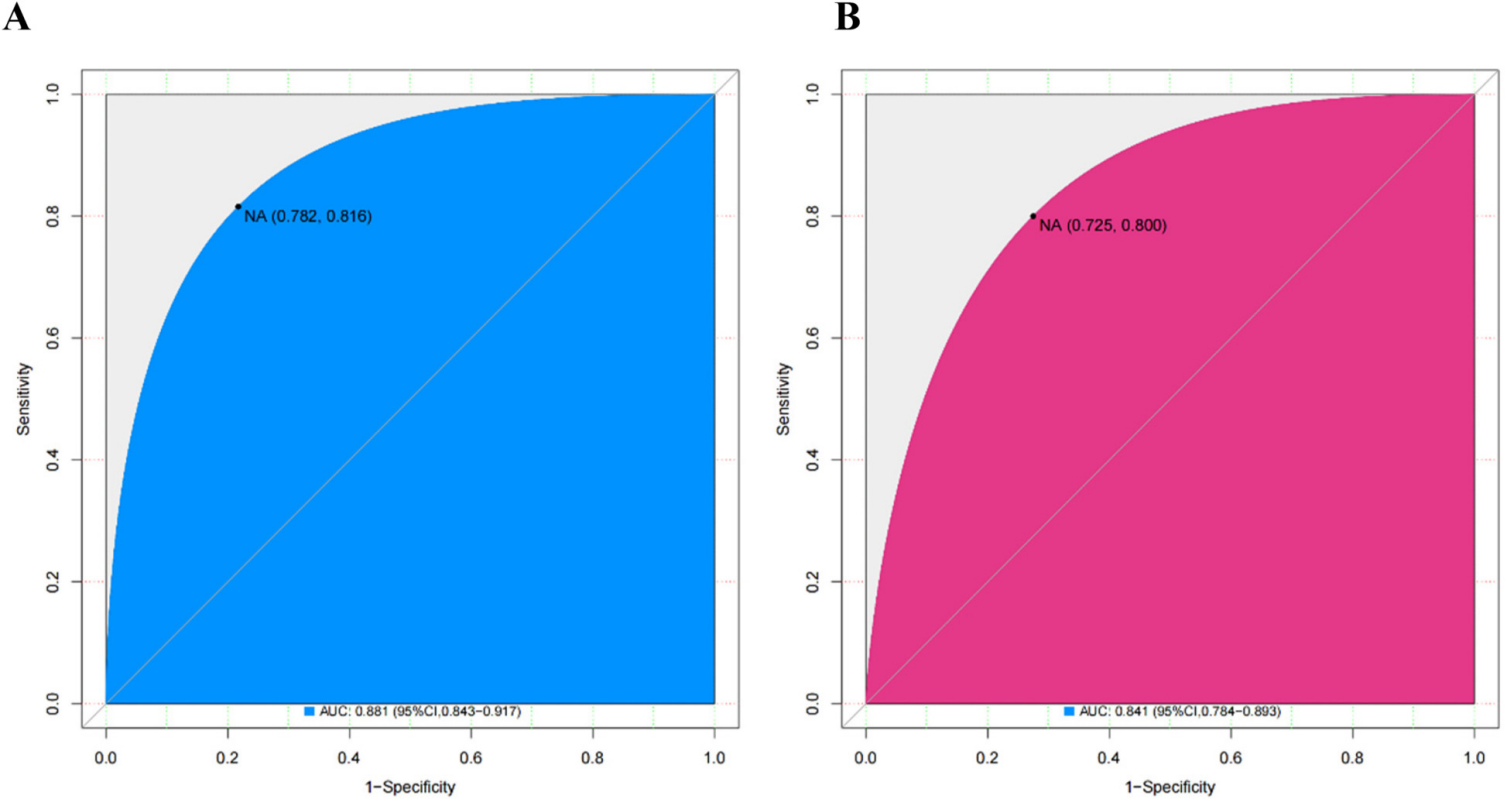
ROC curves for the nomogram in the training cohort **(A)** and the validating cohort **(B)** ROC, receiver operating characteristics.

**Figure 5:**
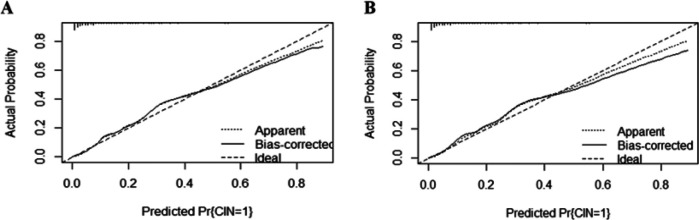
The calibration plot for the nomogram in the training cohort **(A)** and the validating cohort **(B)**. The predicted probability of GIB is plotted on the *x*-axis and the actual probability is plotted on the *y*-axis. CIN, contrast-induced nephropathy.

The area under the curve (AUC) for the nomogram was recorded at 0.881 (95% CI: 0.782–0.816) for the training cohort, as depicted in Figure 4A, and 0.841 (95% CI: 0.725–0.800) for the validating cohort, shown in Figure 4B, according to the receiver operating characteristic (ROC) analysis, indicating excellent discriminative performance. The nomogram's prediction of the probability of CIN after PCI in STEMI patients was consistent with the probability of occurrence in both the training and validation cohorts, as demonstrated by the Hosmer-Lemeshow test (both *p* > 0.05). Calibration plots showed high agreement between the nomogram (Figure 5A) and the validation cohort (Figure 5B).

### Clinical use

**Figure 6:**
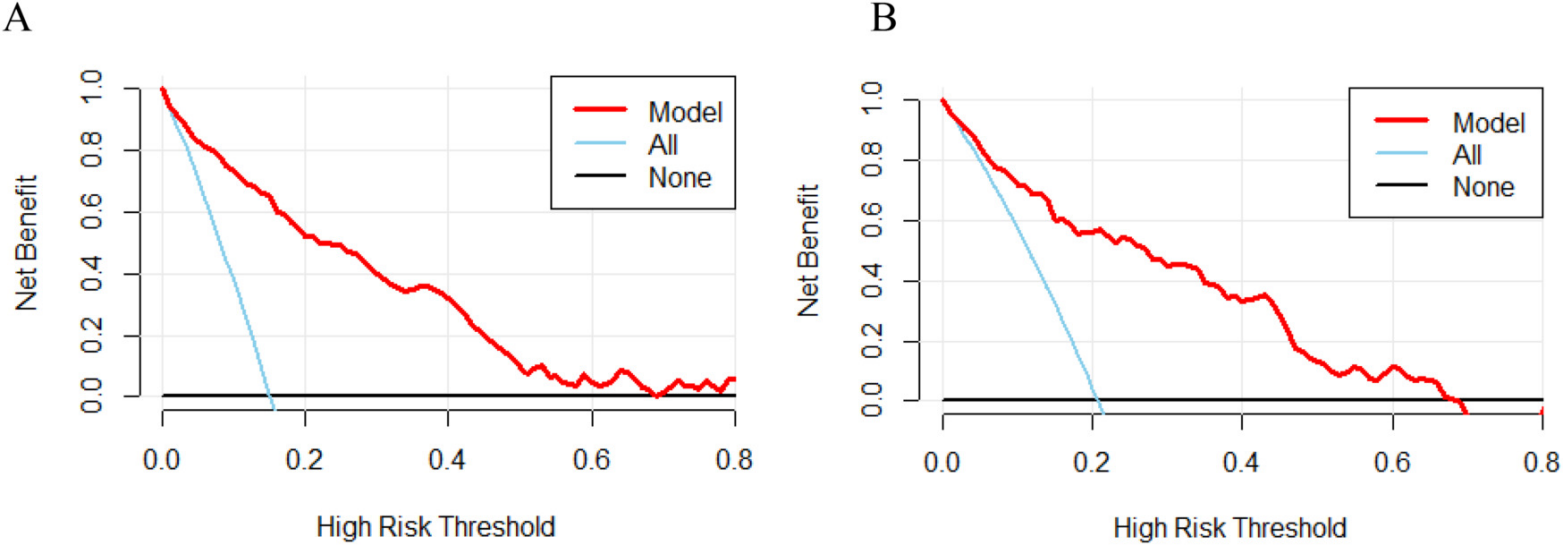
Decision curve analysis (DCA) of nomograms in the training cohort **(A)** and validation cohort **(B)** the bottom black line indicates no intervention and zero net benefit, and the solid blue line indicates an intervention for all patients. The top half of the solid blue line represents positive benefit, and the red line represents the model's critical value.

The decision curve analysis (DCA) was employed to evaluate the applicability and utility of the model in both datasets. The nomograms demonstrated excellent clinical applicability when the threshold probabilities in the training and validation cohorts ranged between 0.05 and 0.70 (Figures 6A,B). This demonstrates a high degree of concordance between the actual distribution and the distribution predicted by the model.

## Discussion

Currently, in clinical practice, PCI remains the primary treatment for myocardial ischemia-reperfusion in patients with ACS, but there is a potential risk of CIN after PCI, which significantly affects the prognosis and quality of life of patients (20). It has been shown that the incidence of CIN is significantly increased in specific groups of high-risk patients such as CKD, diabetes mellitus, and heart failure (21, 22). Therefore, it is important to identify important indicators of CIN and to prevent and intervene in the risk of developing CIN in various ways. Presently, we conducted a retrospective study combining a classical regression analysis method with a machine learning model to predict the risk of CIN in patients with STEMI after PCI by constructing and validating a column chart based on the optimal combination of predictors.

The inclusion of the AMR in our prediction model is an innovative aspect of this paper. AMR has been validated as a reliable indicator of microvascular function and correlates with outcomes of various cardiovascular interventions, providing a more nuanced understanding of microvascular health. This understanding is critical for assessing the risk of CIN after PCI in patients with STEMI. Microvascular dysfunction, as measured by AMR, plays a crucial role in the pathophysiological course of myocardial injury following PCI. Notably, prior studies have shown that impaired microcirculation can extend to renal function, contributing to the development of CIN (22). Elevated AMR values reflect microvascular dysfunction characterized by impaired endothelial function and reduced capillary density, which are often not assessed by traditional risk factors, such as CKD (23, 24). The interconnectedness of the heart and kidneys through systemic circulation means that inadequate renal perfusion can occur during contrast administration, potentially leading to renal injury. This impairment of the microcirculation is particularly deleterious in the setting of STEMI, where the need for effective blood flow is dramatically increased. Research by Guo Z et al. demonstrates that impaired microvascular responses are significantly associated with subsequent renal injury and higher AMR values following PCI (11). Moreover, AMR serves as a predictor of microvascular injury, and high AMR values are strongly associated with an increased incidence of microvascular injury after hemodialysis reconstruction procedures. This injury may exacerbate the toxic effects of contrast media on the kidneys, thereby increasing the risk of nephropathy (25). Clinically, clinicians may consider interventions aimed at improving post-PCI microvascular function in patients identified as high risk with an AMR >2.5. These strategies could include administering medications that enhance endothelial function or employing mechanical devices to optimize microvascular perfusion. Thus, by focusing on improving microcirculation, we may reduce the risk of CIN while maintaining diagnostic accuracy during contrast administration (26).

The TyG index has recently been identified as a marker of insulin resistance (IR) and has been demonstrated to be a more accurate predictor of metabolic disease than the homeostasis model assessment of insulin resistance (HOMA-IR). IR may precede the development of diabetes and coronary artery disease (CAD). Adults with IR are at an increased risk for atherosclerosis and may develop CAD due to metabolic abnormalities. Hu et al. found that a higher TyG index was associated with a poorer prognosis after PCI in patients with ACS (27). Additionally, a retrospective cohort study found that patients with non-ST-elevation ACS (NSTE-ACS) with a high TyG index were significantly associated with a heightened risk of coronary ischemia (CIN), regardless of diabetes status (28). The reason for this may be that the systemic microangiopathy caused by IR has a negative impact on patient prognosis, and further studies are needed to determine whether the TYG index can be generalized to post-PCI complications.

In patients with low eGFR, renal blood flow is often compromised, leading to a state of chronic hypoperfusion (29). The introduction of contrast agents further exacerbates this hypoperfusion by inducing renal cortical/medullary ischemia-hypoxia, serving as a central CIN mechanism through both diminished glomerular filtration and activation of the injurious cellular cascade. Moreover, contrast media promote oxidative stress through the generation of reactive oxygen species (ROS) (30). This oxidative load overwhelms renal antioxidant defenses, resulting in cellular damage and apoptosis, and is closely linked to inflammation, which promotes endothelial dysfunction and fibrosis. Additionally, contrast agents can exert direct nephrotoxic effects, particularly in patients with pre-existing renal impairment, causing tubular cytotoxicity that leads to cell necrosis and shedding into the tubular lumen. This can create obstructions within the tubular system, exacerbating the reduction in effective glomerular filtration (31). The use of certain contrast agents can lead to impaired renal microcirculation, resulting in decreased renal perfusion and filtration capacity. The study conducted by Kulkarni CS et al. confirms that eGFR and contrast volume are the main predictors of CIN in their research (32).

Prior research has indicated a potential correlation between uric acid and CIN (33). Elevated uric acid levels may be indicative of underlying metabolic disturbances and increased inflammatory responses. It has been demonstrated that uric acid has a detrimental impact on cardiovascular health, leading to endothelial dysfunction and oxidative stres (34). The underlying mechanism may be that in patients with STEMI, this effect increases the risk of tubular and glomerular injury, thereby exacerbating the development of CIN. Furthermore, although high-density lipoprotein cholesterol (HDL-C) is protective, its function may be suppressed in the current pathological state, thereby reducing its protective capacit (19). By monitoring UHR and HDL-C levels, clinicians can identify patients at high risk of renal complications at an earlier stage, thereby enabling the implementation of proactive measures to mitigate the risks associated with the administration of contrast agents. Further research is required to ascertain the general applicability of UHR in patients with different types of cardiovascular disease. Furthermore, clinical trials are required to substantiate the potential of UHR as a therapeutic target for the enhancement of renal prognosis subsequent to contrast-induced injury.

In summary, we found that eGFR, AMR, UHR, TYG, and Contrast agent dosage were independent indicators for CIN. First, we obtained more accurate results using a combination of univariate analysis and machine learning. Second, the nomogram model is a scoring system that can be effectively used for the prediction of CIN incidence in STENI patients without the need for complex formula calculations. Third, the model of the relationship between risk factors and CIN is presented graphically, which has the effect of simplicity and intuition, and the results of the study show that our prediction model has good performance. Notably, the incidence of CIN observed in our validation cohort was more than 20%, which is significantly higher than what is commonly reported in the literature. This difference may be due to the fact that our cases may have experienced referral bias or included patients with more complex patient populations.

## Limitations

Although our study contributes to the field, it is important to acknowledge its limitations. As previously mentioned, the retrospective design may introduce selection bias and limit causal inferences. Additionally, our study population was limited to a single center and may not be fully representative of the broader population of STEMI patients, thus requiring validation in a different cohort. Furthermore, data on prophylactic interventions (e.g., hydration protocols, use of N-acetylcysteine) for the prevention of CIN were not available, which may have impacted the observed incidence rates of CIN. The findings may have limited generalizability to STEMI patients and may not apply to other patient populations or clinical settings. Additional research is necessary to evaluate the effectiveness of AMR in predicting contrast nephropathy in various patient populations. Based on Osamu Kurihara et al.'s proposal that microvascular dysfunction may worsen renal hypoperfusion and ischemia following contrast exposure (35), future studies should investigate potential mechanistic pathways connecting AMR to contrast nephropathy.

## Conclusion

The AMR-based predictive model for post-PCI contrast nephropathy in STEMI patients is a significant advancement in understanding and mitigating this complication. By integrating a wide array of variables, including AMR, our model not only enhances predictive accuracy but also provides a valuable tool for developing personalized postoperative treatment plans for patients.
